# Survival Analysis in Patients with Pancreatic Ductal Adenocarcinoma Undergoing Chemoradiotherapy Followed by Surgery According to the International Consensus on the 2017 Definition of Borderline Resectable Cancer

**DOI:** 10.3390/cancers10030065

**Published:** 2018-03-05

**Authors:** Aoi Hayasaki, Shuji Isaji, Masashi Kishiwada, Takehiro Fujii, Yusuke Iizawa, Hiroyuki Kato, Akihiro Tanemura, Yasuhiro Murata, Yoshinori Azumi, Naohisa Kuriyama, Shugo Mizuno, Masanobu Usui, Hiroyuki Sakurai

**Affiliations:** Hepatobiliary Pancreatic and Transplant Surgery, Mie University School of Medicine, 2-174 Edobashi, Tsu, Mie 514-8507, Japan; shujiisaji1@mac.com (S.I.); kishiwad@clin.medic.mie-u.ac.jp (M.K.); t-fujii@clin.medic.mie-u.ac.jp (T.F.); uskm007@clin.medic.mie-u.ac.jp (Y.I.); khmnh0610@clin.medic.mie-u.ac.jp (H.K.); iorichan@clin.medic.mie-u.ac.jp (A.T.); yasumura@clin.medic.mie-u.ac.jp (Y.M.); azu1121@clin.medic.mie-u.ac.jp (Y.A.); naokun@clin.medic.mie-u.ac.jp (N.K.); mizunos@clin.medic.mie-u.ac.jp (S.M.); m-usui@clin.medic.mie-u.ac.jp (M.U.); hirodon@clin.medic.mie-u.ac.jp (H.S.)

**Keywords:** portal vein, superior mesenteric artery, CA 19-9, lymph node metastasis, performance status

## Abstract

*Background*: The aim of this study was to validate a new definition of borderline resectable pancreatic ductal adenocarcinoma (PDAC) provided by the 2017 international consensus on the basis of three dimensions of anatomical (A), biological (B), and conditional (C) factors, using the data of the patients who had been registered for our institutional protocol of chemoradiotherapy followed by surgery (CRTS) for localized patients with PDAC. *Methods*: Among 307 consecutive patients pathologically diagnosed with localized PDAC who were enrolled in our CRTS protocol from February 2005 to December 2016, we selected 285 patients who could be re-evaluated after CRT. These 285 patients were classified according to international consensus A definitions as follows: R (resectable; n = 62), BR-PV (borderline resectable, superior mesenteric vein (SMV)/portal vein (PV) involvement alone; n = 27), BR-A (borderline resectable, arterial involvement; n = 50), LA (locally advanced; n = 146). Disease-specific survival (DSS) was analyzed according to A, B (serum CA 19-9 levels and lymph node metastasis diagnosed by computed tomography findings before CRT), and C factors (performance status (PS)) factors. *Results*: The rates of resection and R0 resection were similar between R (83.9 and 98.0%) and BR-PV (85.2 and 95.5%), but much lower in BR-A (70.0 and 84.8%) and LA (46.6 and 62.5%). DSS evaluated by median survival time (months) showed a similar trend to surgical outcomes: 33.7 in R, 27.3 in BR-PV, 18.9 in BR-A and 19.3 in LA, respectively. DSS in R patients with CA 19-9 levels > 500 U/mL was significantly poorer than in patients with CA 19-9 levels ≤ 500 U/mL, but there were no differences in DSS among BR-PV, BR-A, and LA patients according to CA 19-9 levels. Regarding lymph node metastasis, there was no significant difference in DSS according to each resectability group. DSS in R patients with PS ≥ 2 was significantly worse than in patients with PS 0-1. *Conclusions*: The international consensus on the definition of BR-PDAC based on three dimensions of A, B, and C is useful and practicable because prognosis of PDAC patients is influenced by anatomical factors as well as biological and conditional factors, which in turn may help to decide treatment strategy.

## 1. Introduction

Pancreatic ductal adenocarcinoma (PDAC) is known to be a systemic disease at the time of diagnosis because approximately 30% of patients among those who undergo surgical resection die of the disease within 1 year after surgery [1,2,3]. Survival rate in the subgroup showing early recurrence are comparable to those observed in patients with advanced disease undergoing antitumoral therapies alone [4]. Therefore, for the patients with PDAC, a multimodal treatment is required from the time of diagnosis because surgical treatment alone does not greatly improve survival. During the last decade, however, there has been an improvement in survival in patients with PDAC due to advances in anticancer therapy as well as an improvement in patient selection by pancreatic surgeons [5,6,7].

To improve patient selection for surgery according to the likelihood of an R0 resection, since 2006 the National Comprehensive Cancer Network (NCCN) has developed guidelines to define tumor resectability in PDAC. Using their criteria, PDAC is classified as resectable (R), borderline resectable (BR), or unresectable (UR), which includes locally advanced (LA) or metastatic disease. Although BR-PDAC can be defined as disease with an increased likelihood of an incomplete resection, its definition has been a much debated issue. Several different versions have been proposed over the years, all of them based on the extent of vessel involvement (venous and arterial) by the tumor [8,9,10]. The main definition of BR pancreatic cancer refers to the concept of technical resectability, but also a high risk of positive surgical margins and recurrence, raising questions about the real value of surgery and suggesting a benefit of preoperative treatment in this subset of patients. This highlights the substantial need to expand the concept of what defines BR-PDAC. In particular, current definitions of R, BR, and UR are based only on radiological parameters and do not take into consideration the biology of the disease.

In clinical practice, however, the surgeon’s decision on PDAC resection is not based solely on anatomic criteria. The biological behavior of PDAC is an important consideration and will become more important with accumulated knowledge of the genomic basis for cancer invasion and metastases. Another important consideration, there is the conditional status of the host, that is, the ability of the patient to withstand the physiological challenge of surgery. Although biological and conditional criteria for resectability were first reported by Katz et al. in 2008 [11], they have not been incorporated into the later definitions of BR-PDAC. To address these issues and to seek an international consensus on BR-PDAC, a symposium was held during the 20th meeting of the International Association of Pancreatology (IAP) in Sendai, Japan, in 2016, where the presenters sought international consensus on issues related to BR-PDAC. As a result, they defined patients with BR-PDAC according to the three distinct dimensions: anatomical (A), biological (B), and conditional (C) [12]. The A definition of BR-PDAC is a tumor that is at high risk for margin-positive resection when surgery is used as an initial treatment strategy. Neoadjuvant chemotherapy and/or radiotherapy are considered to increase the chance of a R0 resection. The B definition is the presence of findings that increase the possibility of extra-pancreatic metastatic disease in potentially resectable disease based on anatomic criteria. The C definition is when the patient has a high risk for morbidity or mortality after surgery because of host-related factors including performance status and comorbidities in potentially resectable disease based on anatomic criteria.

Our institution has performed chemoradiotherapy followed by surgery (CRTS) for the treatment of localized PDAC since February 2005 in attempt to improve patient survival as a prospective study, in which we have found that anatomical definition of R, BR, and LA previously reported can predict patient survival very well by using data of registered patients with and without resection [13,14,15,16,17]. The aim of this study was to validate a new definition of borderline resectable PDAC provided by the 2017 international consensus, using the data of the patients who had been registered for our institutional protocol of CRTS.

## 2. Materials and Methods

**Figure 1:**
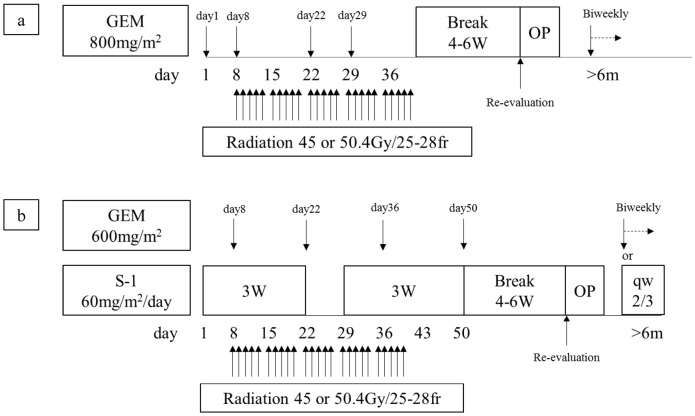
The CRTS protocol at Mie University Hospital. (**a**) GEM-CRT (2005.2–2011.10, enrolled patients: n = 128, re-evaluated patients: n = 116); (**b**) S-1/GEM-CRT (2011.11–2016.12, enrolled patients: n = 179, re-evaluated patients: n = 169). GEM: gemcitabine, W: week, OP: operation, m: months, qw: every week, CRTS: chemoradiotherapyfollowed by surgery.

Between February 2005 and December 2016, we had enrolled 307 patients who were cytologically or histologically diagnosed as having localized PDAC determined by using 64-slice multidetector computed tomography (MDCT) for our CRTS protocol reported previously [15,17]. CT was performed according to a defined pancreas protocol as four-phasic contrast-enhanced MDCT with thin slices at intervals of 1 mm. Patients were excluded when they showed evident distant metastatic lesions at the time of enrollment. All patients gave their written informed consent for inclusion in the study. The study protocol was approved by the medical ethics committee of Mie University Hospital (No. 2955), and the study was performed in accordance with the ethical standards established in the 1964 Declaration of Helsinki. Our treatment protocol for gemcitabine-based CRT (GEM-CRT) was used to treat 128 PDAC patients from February 2005 to October 2011. Thereafter, we switched our protocol to S-1/GEM-based CRT (S-1/GEM-CRT), which was used to treat 179 patients from November 2011 to December 2016 (Figure 1). Although we initially referred to our protocol as neoadjuvant or preoperative CRT [13,14], we subsequently adopted the term “CRTS,” because approximately half of the patients registered in the protocol were staged as having locally unresectable PDAC for these patients, the term “preoperative treatment”, was not appropriate for these patients [15,17].

For the present study, at the initial visit to our hospital all of these 307 patients were reclassified into the four groups (R, BR-PV, BR-A, and LA) according to international consensus of classification of resectability based on anatomical definitions using CT imaging including coronal and sagittal sections [12] at the initial visit to our hospital. The CT criteria of the internal consensus are as follows. Resectable (R) criteria are (1) superior mesenteric vein (SMV)/portal vein (PV), no tumor contact or unilateral narrowing; and (2) superior mesenteric artery (SMA), celiac artery (CA), and common hepatic artery (CHA), no tumor contact. BR (borderline resectable)-PDAC is subclassified into BR-PV (SMV/PV involvement alone) and BR-A (arterial involvement). BR-PV is defined by (1) SMV/PV, tumor contact 180 degrees or greater or bilateral narrowing/occlusion, not exceeding the inferior border of the duodenum; and (2) SMA, CA, CHA, no tumor contact/invasion. BR-A is defined by (1) SMA, CA, tumor contact of less than 180 degrees without showing deformity/stenosis; and/or (2) CHA, tumor contact without showing tumor contact of the proper hepatic artery (PHA) and/or CA. Unresectable (UR)-PDAC is subclassified as locally advanced or metastatic according to the status of distant metastasis as follows. Locally advanced (LA) is defined as (1) SMV/PV bilateral narrowing/occlusion, exceeding the inferior border of the duodenum; (2) SMA, CA tumor contact/invasion of 180 or more degree; (3) CHA tumor contact/invasion showing tumor contact/invasion of the PHA and/or CA; or (4) aorta (AO) tumor contact or invasion. Metastatic (M) is defined as the presence of distant metastasis including macroscopic para aortic and extra abdominal lymph node metastasis. 

**Figure 2:**
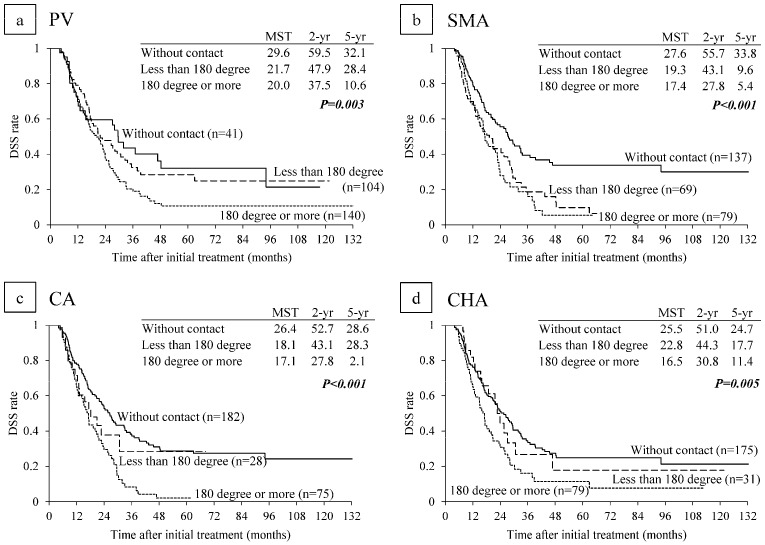
Disease-specific survival (DSS) curves according to major vessel contact/invasion on CT image before CRT. (**a**) PV (Portal vein); (**b**) SMA (Superior mesenteric artery); (**c**) CA (Celiac artery); (**d**) CHA (Common hepatic artery). MST: median survival time (months).

Table 1 shows the characteristics and backgrounds of the 307 enrolled PDAC patients, according to international consensus of classification of resectability based on anatomical definition: R in 69 patients, BR-PV in 31, BR-A in 52, and LA in 155. Of the 307 patients, 285 (92.3%) could be re-evaluated after completion of CRT after exclusion of 10 patients with uncompleted CRT, 10 with rejection of re-evaluation, and two who underwent surgery at another hospital (Figure 2). The subjects of the present study were these 285 patients with re-evaluation. Among the 285 patients, 91 (31.9%) were determined inoperable at re-evaluation (87 were non-resected and four underwent palliative resection to control tumor bleeding), and 194 (68.1%) were determined operable. Of the 194 patients, curative-intent resection could be performed in 168 including R0 resection in 137, R1 in 29, and R2 in two patients. Six patients who were diagnosed as having distant metastasis at laparotomy underwent palliative resection because distant metastasis in the liver or peritoneum was not extensive, and the remaining 20 patients were non-resected because of extensive local tumor invasion and/or extensive distant metastases at laparotomy.

## 3. Evaluation of Biological and Conditional Factors

According to international consensus [12], the biological definition of BR-PDAC is a tumor that is potentially resectable anatomically with clinical findings suspicious of distant metastasis, including CA 19-9 level > 500 U/mL, or regional lymph node metastasis diagnosed by biopsy or PET-CT but distant metastasis not proven. In the present study, serum CA 19-9 levels were measured immediately before the initiation of CRT. In the patients with obstructive jaundice, drainage was achieved before CRT using endoscopic retrograde biliary drainage, endoscopic nasobiliary drainage, or percutaneous transhepatic cholangiodrainage. CA 19-9 level may not be an accurate reflection of disease status in patients who express the Lewis (a-b-) genotype [18]. However, as genotyping was not performed routinely, and thus, we could not determine the patients with Lewis (a-b-) but these patients usually presented with values lower than the assay sensitivity threshold (1 U/mL). Consequenetly, the 13 patients with CA 19-9 ≤ 1 U/mL were excluded from the evaluation of CA 19-9.

Because regional lymph node metastasis could not be diagnosed by biopsy or PET-CT in the present study, lymph node metastasis was determined according to diagnostic CT criteria for lymph node metastasis from the Classification of Pancreatic Cancer by Japan Pancreas Society (JPS) (4th English Edition) [10]. On dynamic MDCT, an enlarged node of more than 10 mm in the shorter diameter that included a low absorption area, suggesting an area of necrosis, was diagnosed as metastasis. Regional lymph node metastasis on CT imaging was classified according to UICC TNM classification of malignant tumor (8th edition) as follows: N0 (no regional lymph node metastasis), N1 (metastases in one to three regional lymph nodes), and N2 (metastases in four or more regional lymph nodes) [19]. According to the international consensus [12], patients with anatomically resectable PDAC and with performance status (PS) ≥ 2 are defined as BR-PDAC. Just before the initiation of CRT, PS was recorded using the Zubrod/Eastern Cooperative Oncology Group (ECOG) scale [20]. 

## 4. Indication of Curative-Intent Resection, Surgical Procedure, and Postoperative Complications 

At the time of reassessment after CRT, the patients in whom down-staging from LA to BR was achieved were planned to undergo surgery. Among the patients in whom down-staging was not achieved, those with no encasement and deformity of major arteries such as CA, SMA, or jejunal arteries were offered the surgery. Normalization or remarkable decrease in serum value of CA 19-9 was a criteria for decision of surgery. At laparotomy, it was mandatory for the resection candidate to have no liver metastasis and peritoneal dissemination. Staging laparoscopy was not routinely performed, and washing cytology was usually performed but its result was not used to decide the indication of resection. Pancreaticoduodenectomy (PD) was performed with nerve plexus hanging maneuver using anterior approach, which helps to secure a negative surgical margin around the SMA [21]. Distal pancreatectomy (DP) was performed using posterior radical antegrade modular pancreatosplenectomy (posterior RAMPS) procedure, which makes it possible to secure negative tangenital margin [22]. If the tumor has invasion to celiac axis, distal pancreatectomy with en block celiac axis resection (DP-CAR) is performed [23]. Resection and reconstruction of the PV/SMV were performed when the surgeon could not separate the pancreatic head or the uncinate process from these vessels without leaving gross tumor on the vessel. When limited involvement of the common hepatic artery was identified, a segmental resection of this vessel was performed with primary anastomosis. For lymph node dissection, we did not perform extended lymph node dissection but only dissection regional lymph nodes defined in classification of pancreatic cancer by JPS [10]. The patients who had an unresectable disease at surgery, which was usually due to the presence of distant metastasis, underwent surgical bypass as clinically indicated. 

Postoperative complications including morbidity and mortality were graded according to the Clavien-Dindo classification [24]. Postoperative pancreatic fistula (POPF) was graded according to the International Study Group on Pancreatic Fistula classification [25], and Grades B and C were determined as POPF. Postoperative mortality was defined as all causes of death in the hospital. 

## 5. Statistical Analyses

Continuous variables were expressed as median (range). In all patients who came for reassessment, the date of the initial treatment was chosen as the starting point for the measurement of survival time. Patients who were alive or had died of a cause other than PDAC were censored for analysis of disease-specific survival (DSS). DSS was calculated by the product-limit method of Kaplan and Meier, and survival rates were compared using log-rank tests. The day of final follow-up was 31 July 2017, and there was no loss of follow-up. Median duration of follow-up period was 16.5 months (range: 0.9–134.1) after the initial treatment. All statistical analyses were performed using the SPSS version 18 (SPSS Inc., Chicago, IL, USA) software. A *p* value less than 0.05 was considered statistically significant. 

## 6. Results

### 6.1. Clinical and Surgical Outcomes According to Anatomical Classification of Resectability

Among 285 re-evaluated patients, the resection after CRT was performed in 178 patients (62.5%). Regarding surgical procedures, subtotal stomach preserving pancreatoduodenectomy (SSPPD) was performed in 143 patients, DP in 34 (including DP-CAR in 10), and total pancreatectomy in 1. In the 143 patients who underwent SSPPD, the median operative duration was 561 min (range: 281–842 min), and the median intraoperative blood loss was 1003 mL (range: 80–11,937 mL), and combined resection rate of PV, HA, and CA was 90.9, 5.6, and 0%, respectively. The postoperative complication rate (Clavien-Dindo classification III or greater) was 23.8% and the incidence of POPF was 3.5%, which was much lower than the previously reported incidence because preoperative chemoradiotherapy is known to reduce incidence of POPF [26,27]. In the 34 patients who underwent DP, the median operative duration was 416 min (range: 195–830 min), and the median intraoperative blood loss was 877 mL (range: 110–5033 mL), and combined resection rate of PV, HA, and CA was 38.2, 29.4, and 29.4%, respectively. The postoperative complication rate (Clavien-Dindo classifi-cation III or greater) was 35.3% and the incidence of POPF was 17.6%.

R0 resection was achieved in 137 (81.5%) patients among the 168 patients, excluding four who underwent palliative pancreatectomy despite the presence of distant metastases at re-evaluation and six who underwent pancreatectomy despite of the presence of distant metastases at laparotomy. In each resectability group (R, BR-PV, BR-A, and LA), the resection rate was 83.9, 85.2, 70.0, and 46.6%, and R0 resection rate 98.0, 95.5, 84.8, and 62.5%, respectively. The rate of metastasis occurrence within 3 months from initial treatment was 9.7, 11.1, 10.0, and 11.0%, respectively (Table 2). There are no 30-day postoperative deaths and hospital death occurred in three patients. The median length of hospital stay was 43 days (range: 15–205 days). 

As of the difference of prognosis between GEM- and S-1/GEM-CRT, MST was significantly longer in the patients who received S-1/GEM-CRT (n = 116) than in those who received GEM-CRT (n = 169): 23.9 and 15.5 months (*p* = 0.007).

### 6.2. Survival Analysis According to Major Vascular Invasion on CT

DSS was compared according to clinical major vascular contact/invasion on CT imaging before CRT. For SMV/PV contact/invasion, median survival time (MST) was the longest in the patients without contact, followed by those with invasion less than 180 degrees and those with invasion of 180 degrees or more (29.6, 21.7, and 20.0 months respectively, *p* = 0.003). DSS curves between the patients without contact and those with less than 180 degrees were very similar (5-year DSS rate: 32.1% and 28.4%), but the patients with SMV/PV contact of 180 degrees or more had significantly poorer survival (5-year DSS rate: 10.6%) (Figure 2a). For SMA contact/invasion, MST was the longest in the patients without contact, followed by those with invasion less than 180 degrees and 180 degrees or more (27.6, 19.3, and 17.4 months respectively, *p* < 0.001). DSS curves between SMA contact less than 180 degrees and SMA contact of 180 degrees or more were very similar (Figure 2b). For CA contact/invasion, MST was the longest in the patients without contact followed by those with invasion less than 180 degrees and those with invasion of 180 degrees or more (26.4, 18.1, and 17.1 months respectively, *p* < 0.001). The 5-year DSS rate in patients with CA contact less than 180 degree was 28.3%, which was much higher than the rate of 2.1% in patients with CA contact of 180 degrees or more (Figure 2c). For CHA contact/invasion, MST was the longest in the patients without contact, followed by those with invasion less than 180 degrees and those with invasion of 180 degrees or more (25.5, 22.8, and 16.5 months respectively, *p* = 0.005). DSS curves between the patients without CHA contact and those with CHA contact less than 180 degree were similar (5-year DSS rate: 24.7 and 17.7%), but the patients with CHA contact of 180 degrees or more had much poorer survival (5-year DSS rate: 11.4%) (Figure 2d).

### 6.3. Survival Analysis According to Anatomical Resectability Classification

**Figure 3:**
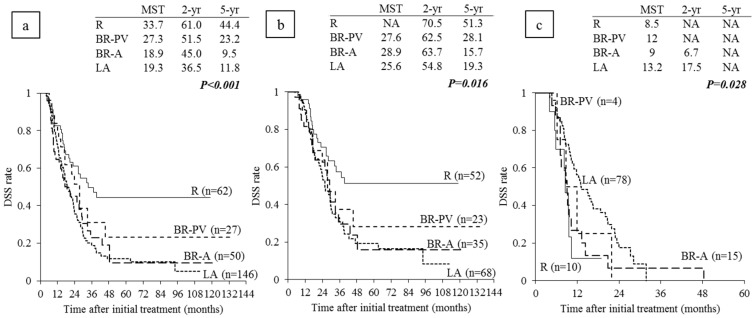
Disease-specific survival (DSS) curves according to the anatomical resectability classification. (**a**) All patients who completed CRT and underwent re-evaluation after CRT (n = 285); (**b**) Patients with resection * (n = 178); (**c**) Patients without resection (n = 107). * Including four patients with distant metastasis at re-evaluation (two in R and two in BR-A) and six patients with intraabdominal distant metastasis at laparotomy (one in R, one in BR-PV and four in LA). R: resectable, PDAC: pancreatic ductal adenocarcinoma, BR-PV: borderline resectable (superior mesenteric vein/portal vein invasion alone), BR-A: borderline resectable (arterial invasion), LA: locally advanced, MST: median survival time (months), NA: not applicable.

In all 285 re-evaluated patients, MST in R patients was significantly longer than that in BR-PV, BR-A, and LA patients (33.7, 27.3, and 18.9, and 19.3 months, *p* < 0.001; Figure 3a). A similar result was found in the 178 patients with resection: MST in R patients was significantly longer than that in the other BR-PV, BR-A, and LA patients (NA, 27.6, 28.9, and 25.6 months. *p* = 0.016; Figure 3b).

There was no significant difference of prognosis between in the patients who were received PD (n = 143) and in those received DP (n = 34). In 143 patients who were received PD, MST in R patients (n = 41) was extremely longer than those in the other BR-PV (n = 22), BR-A (n = 30), and LA (n = 50) patients: NA, 27.6, 28.9, and 25.6 months (*p* = 0.020), however, in 34 patients who were received DP, MST was compatible in R (n = 11), BR-A (n = 5), and LA (n = 18) patients: 37, NA, and 21.7 months (*p* = 0.723). In the 107 patients without resection, MST in R patients was the shortest, followed by BR-A, BR-PV, and LA (8.5, 9, 12, and 13.2 months, respectively; Figure 3c). The reason why MST in R patients was the shortest was considered to be that all of them had distant metastases at the time of re-evaluation or laparotomy, while some LA patients had been unresectable because of their local anatomical factors alone.

### 6.4. Survival Analysis According to Serum CA 19-9 Levels and Lymph Node Metastasis on CT before CRT

**Figure 4:**
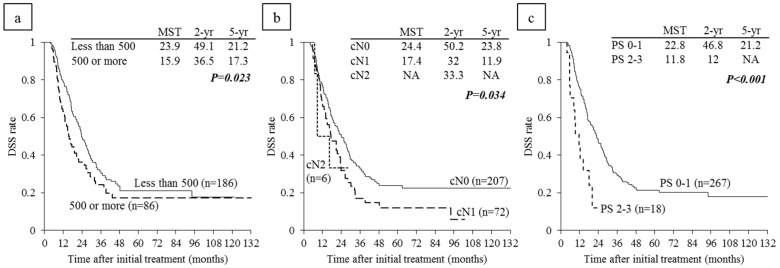
Disease-specific survival (DSS) curves according to (**a**) serum CA 19-9 level * before CRT in the reevaluated patients excluding the 13 patients whose CA 19-9 level was 1 or less U/mL (n = 272); (**b**) according to lymph node metastases on CT before CRT (n = 285); and (**c**) performance status (PS) before CRT (n = 285). * Cut-off value of serum CA 19-9 level before CRT is 500 U/mL. CRT: chemoradiotherapy, NA: not applicable.

Among 272 re-evaluated PDAC patients, excluding the 13 patients whose CA 19-9 level before CRT was ≤1 U/mL, DSS was compared between high and low CA 19-9 levels. When the cut-off value of serum CA 19-9 level before CRT was set at 500 U/mL, the patients with CA 19-9 level ≤ 500 U/mL had significantly longer MST than those with CA 19-9 levels > 500 U/mL (*p* = 0.023) (Figure 4a). MST in the 207 patients without lymph node metastases evaluated on CT (cN0) was significantly longer than that patients with lymph node metastases (cN1 and cN2): 24.4 vs. 17.4 and NA (*p* = 0.034) (Figure 4b).

**Figure 5:**
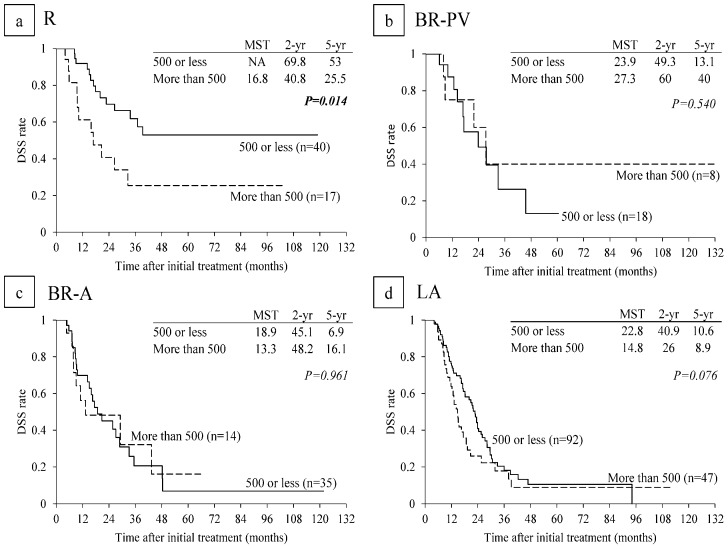
Disease-specific survival (DSS) curves according to serum CA 19-9 level in (**a**) R (n = 57); (**b**) BR-PV (n = 26); (**c**) BR-A (n = 49); and (**d**) LA (n = 139) in the re-evaluated patients excluding the 13 patients whose CA 19-9 level was 1 or less U/mL (n = 272) (cut-off value of CA 19-9 is 500 U/mL). R: resectable, BR-PV: borderline resectable (superior mesenteric vein/portal vein invasion alone), BR-A: borderline resectable (arterial invasion), LA: locally advanced, MST: median survival time (months), NA: not applicable.

**Figure 6:**
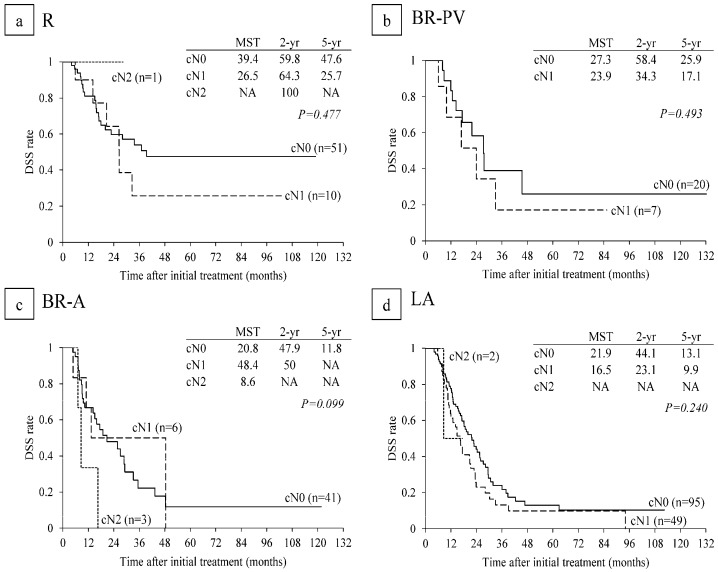
Disease-specific survival (DSS) curves according to lymph node metastaseon CT before CRT in (**a**) R (n = 62); (**b**) BR-PV (n = 27); (**c**) BR-A (n = 50); and (**d**) LA (n = 146). R: resectable, BR-PV: borderline resectable (superior mesenteric vein/portal vein invasion alone), BR-A: borderline resectable (arterial invasion), LA: locally advanced, MST: median survival time (months), NA: not applicable.

In subanalysis of each resectability group, MST in R patients with CA 19-9 level ≤ 500 U/mL was significantly longer than in R patients with CA 19-9 level > 500 (NA vs. 16.8 months: *p* = 0.014) (Figure 5a). MSTs in BR-PV and BR-A patients with CA 19-9 level ≤ 500 U/mL did not differ significantly from those in patients with CA 19-9 level > 500 U/mL (Figure 5b,c). MST in LA patients with CA 19-9 level ≤ 500 tended to be longer than in patients with CA 19-9 level > 500 U/mL (22.8 vs. 14.8 months: *p* = 0.076) (Figure 5d). Regarding lymph node metastasis, there were no significant differences in DSS times among cN0, cN1, and cN2 (Figure 6).

### 6.5. Survival Analysis According to Performance Status

**Figure 7:**
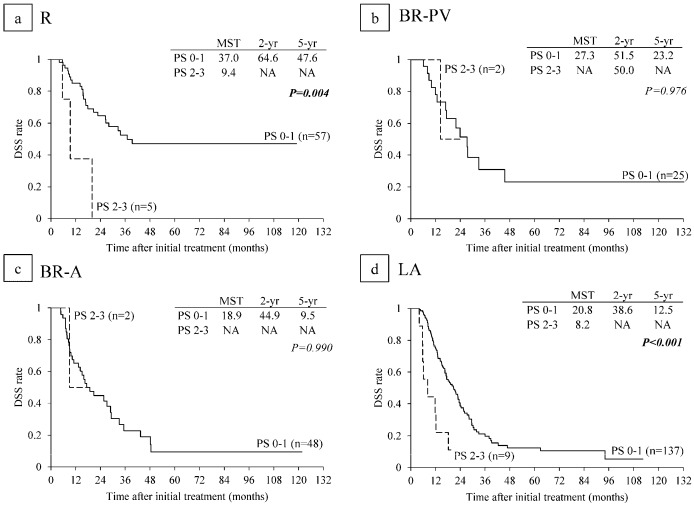
Disease-specific survival (DSS) curves according to performance status (PS) in (**a**) R (n = 62), (**b**) BR-PV (n = 27); (**c**) BR-A (n = 50); and (**d**) LA (n = 146). R: resectable, BR-PV: borderline resectable (superior mesenteric vein/portal vein invasion alone), BR-A: borderline resectable (arterial invasion), LA: locally advanced, MST: median survival time (months), NA: not applicable.

MST in the 267 patients with PS 0-1 was significantly longer than that in 18 patients with PS 2-3 (22.8 vs. 11.8: *p* < 0.001) (Figure 4c). Among the 18 patients with poor PS, 12 patients were PS 2 and six patients were in PS 3. In subanalysis of each resectability group, MST in PS 0-1 patients was significantly longer than that in PS 2-3 in R and LA subgroups (*p* = 0.004, *p* < 0.001) but there were no significance differences in BR-PV and BR-A (Figure 7).

## 7. Discussion

In the present study we used our institutional data on 285 patients with re-evaluation after CRT to validate the international consensus on the definition of BR-PDAC according to the three distinct dimensions of A, B, and C. Our data showed that surgical outcomes evaluated by rates of resection and R0 resection were similar between R (83.9% and 98.0%) and BR-PV (85.2% and 95.5%), but much lower in BR-A (70.0% and 84.8%) and LA (46.6% and 62.5%). Survival analysis evaluated by MST in months showed the trends similar to surgical outcomes: 33.7 months in R, 27.3 in BR-PV, 18.9 in BR-A, and 19.3 in LA. For the B aspect, prognosis in R patients with CA 19-9 levels > 500 U/mL was significantly poorer than in those with CA 19-9 levels ≤ 500 U/mL, whereas there were no survival differences among BR-PV, BR-A, and LA patients according to CA 19-9 levels. For the C aspect, prognosis in R patients with PS 2 or higher was significantly poorer than in those with PS 0-1.

Several different versions of resectability classification for localized PDAC have been reported over the years, but all of them are based on the extent of major vessel involvement by the tumor [8,9,10]. According to previous radiographic definitions on CT, the degree of interface or contact between tumor and vessels has been defined as less than 180 degrees or 180 degrees or more. According to survival analyses in patients with localized PDAC among radiographic PV interface groups on CT (comparing no interface, PV contact less than 180 degrees, and PV contact of 180 degrees or more [28]), the survival curve of patients with no interface was similar to that of patients with PV contact less than 180 degrees, and patients with PV contact of 180 degrees or more had significantly shorter survival than those with PV contact less than 180 degrees. Our present study on CT radiographic PV contact showed similar results: DSS curves between the patients without PV contact and those with contact less than 180 degree were very similar (5-year DSS rate: 32.1% and 28.4%), but the patients with PV contact of 180 degrees or more had significantly poorer survival (5-year DSS rate: 10.6%). Recently, Yamada et al. [29] evaluated performed analysis according to invasion of major vessels, which was incorporated into the definition of resectability classification by JPS (10), using their institutional data of 382 consecutive patients who underwent curative resection for PDAC by excluding patients with neoadjuvant chemotherapy or CRT. The MST in R patients was 30.5 months, which was significantly better than value of 20.5 months in PV (+), 15.8 in CHA (+), and 13.6 in SMA (+). Univariate analysis revealed that PV contact of 180 degrees or more, SMA/CA contact less than 180 degrees, and SMA/CA contact 180 degrees or more were significant prognostic factors, but CHA contact was not a significant factor. In their study, the patient cohort was selected only from patients who underwent curative resection, including only 7.6% (29/382) with LA-PDAC, and furthermore SMA and CA contacts were not separately analyzed. Our data were very similar to those of Yamada et al. with the exception of CHA, which was not a significant prognostic factor in their study [29].

Resectability classification by IAP international consensus 2017 [12] is very similar to that of JPS [10]. Yamada et al. [29] reported that MST was 32.1 months in R, 17.3 in BR-PV, 14.3 in BR-A, and 15.8 in LA, and the authors concluded that PDAC patients with BR-PV and BR-A could be managed as a single subset. In our study, however, MST in all 285 registered patients for CRTS, including 107 (37.5%) without resection, was 33.7 in R, 27.3 in BR-PV, 18.9 in BR-A, and 19.3 in LA, thus showing a much shorter MST in BR-A and LA. Resection rates were similar between R and BR-PV were similar (83.9% and 85.2%), but much lower in BR-A and LA (70.0% and 46.6%). However, MST in 178 patients with resection, however, was NA in R, 27.6 months in BR-PV, 28.9 in BR-A, and 25.6 in LA, showing similar survival between BR-PV and BR-A. Kato et al. [30] retrospectively analyzed prognostic factors for 624 BR-PDAC patients whose data were collected by distributing questionnaires to member institutions of the Japanese Society of Pancreatic Surgery in 2010, and revealed that the survival rates were significantly lower in BR-A than in BR-PV. Additionally, multivariate analysis revealed that SMA/CA involvement on CT, preoperative treatment, surgical resection, and postoperative chemotherapy were independent prognostic factors in all patients, whereas SMA/CA involvement on CT and remnant tumor status were independent prognostic factors among the patients with resection. Taking these results together, subclassification of BR-PDAC into BR-PV and BR-A is useful and practicable for deciding the treatment strategy of PDAC. Specifically, it suggests that preoperative therapy for BR-PDAC enhances patient survival and helps to select the patients who can benefit from surgical resection.

The biological definition of BR-PDAC by international consensus [12] is a resectable tumor with clinical findings suspicious of distant metastasis, including CA 19-9 level > 500 U/mL or regional lymph nodes metastasis. Hartwig et al. [31] investigated the correlation between CA 19-9 levels and prognosis in 1600 patients with potentially resectable PDAC and showed that patients with CA 19-9 level > 500 U/mL had significantly poorer survival than those with CA 19-9 level ≤ 500 U/mL. In our study, the patients with CA 19-9 levels ≤ 500 U/mL showed significantly better survival than those with CA 19-9 > 500 U/mL. With regard to each resectability group, CA 19-9 levels significantly affected survival only in R patients, but did not influence survival in BR-PV, BR-A, and LA patients. Therefore, biological definition of BR-PDAC by international consensus can be considered useful and practicable for deciding treatment strategy. In R patients, the indication of resection in patients with CA 19-9 level > 500 U/mL should be more carefully determined by prolonging preoperative therapy, taking CA 19-9 levels into consideration. Regarding regional lymph node metastasis, it is known that the existence of positive node metastasis strongly affects the prognosis of PDAC patients. According to survival analysis of PDAC patients with resection based on data of the Japanese Pancreatic Cancer Registry (from 2001 to 2007, n = 2304) [10,12], overall survival was significantly better in the patients with N0 status, followed by patients with N1 and those with N2, among patients staged as T1, T2, and T3 by UICC (7th edition) respectively. However, in T4 patients, there was no association between positive lymph node and prognosis, suggesting that the positive lymph node is associated with poor prognosis even in the patients with anatomically resectable PDAC. The previous reported data, however, were based on histological examination of lymph nodes retrieved during operation. In the present study, lymph node metastasis was determined by CT criteria, although international consensus 2017 recommends diagnosis of lymph nodes metastasis using biopsy or PET-CT. In our 285 patients, the existence of lymph node metastasis significantly affected patient prognosis, but there were no significant differences in survival according to each resectability group. This may be because lymph node metastasis was determined by CT imaging, and further study is required using PET-CT or lymph node biopsy under endoscopic ultrasound or CT guidance. The biological behavior of PDAC will become a more important consideration with increasing knowledge of the genomic basis of disease, such as expressions of SMAD4 [32], hENT1 [14,16] and Tenascin C [33]. Our previous study [16] revealed that the assessment of hENT1 expression using EUS-FNAB samples before CRT provided important information on the patients with PDAC who could benefit from curative-intent resection, because prognosis was similarly poor between the patients with negative expression of hENT1 who underwent resection and those who could not undergo resection. For future studies, the examination of these biological markers including genomics would contribute to find out a more reliable prognostic indicator for PDAC.

Host-related factors such as patient PS and immunonutritional status are very important when making a decision of surgical resection or chemotherapy because they are associated with intolerance to the neoadjuvant therapy, postoperative morbidity/mortality, and poor overall prognosis. The UICC TNM classification recently published in 2017 adopts ECOG status (PS) as a host-related prognostic risk factor for PDAC. However, PS has not been incorporated into stage classification. Using the data of 335 patients with histologically confirmed diagnosis of PDAC from 2000 to 2010, Tas et al. [34] revealed that PS was significant prognostic factor for all stages of PDAC. In stages I and II, which are comparable to R and BR-PV, MST in patients with PS 0-1 was 23.5 months, which was significantly longer than 12.4 months in those with PS 2 or more. In stage III, which is comparable to BR-A and LA, MST in patients with PS 0-1 was 10.7 months, which was significantly longer than 4.6 months in those with PS 2 or more. In stage IV (distant metastasis), MST in patients with PS 0-1 was 7.4 months, significantly longer than 3.1 months in those with PS 2 or more. Our data from the present study are comparable to those of Tas et al. In R patients, MST for PS 0-1 was 37.0 months, significantly longer than that 9.4 months for PS 2-3. In LA patients, MST for PS 0-1 was 20.8 months, which was significantly longer than that 8.2 months in PS 2-3. However, there were no significant differences in BR-PV and BR-A, probably because there were few patients with PS 2-3. When we consider surgical indications for PDAC, patients with PS 3 or 4 is usually a contraindication regardless of tumor resectability; however, patients with PS 2 are considered candidates to undergo surgical resection because marginal PS might be reversible in some patients who can be targeted for nutritional supports and prehabilitation to prepare for a surgical treatment [35]. In recent years, another conditional host-related factors such as immunonutritional status and systemic inflammatory response have been assumed to be important, such as the modified Glasgow Prognostic Score, neutrophil/lymphocyte ratio [36], and Onodera’s prognostic nutritional index [37]. In the near future, these factors are likely to be included as aspects of the conditional definition of BR-PDAC.

## 8. Conclusions

In conclusion, international consensus on the definition of BR-PDAC based on the basis of three dimensions of A, B, and C is both useful and practicable because prognosis of PDAC patients is influenced by anatomical factors as well as biological and conditional factors, and may help to decide treatment strategy. (1) In the A definition, preoperative treatment such as CRT may enhance R0 resection rate; (2) in the B definition, even if tumor is considered resectable, preoperative treatment is highly recommended to exclude the patients with metastatic disease; and (3) in the C definition, even if tumor is considered resectable, surgery should be postponed until the PS has been improved by nutritional and rehabilitation support.

## Figures and Tables

**Table 1:** Characteristics and backgrounds of the 307 PDAC patients enrolled for CRTS in each resectability group.

Characteristic	R n = 69	BR-PV n = 31	BR-A n = 52	LA n = 155	All n = 307
Age (year old)	68 (47–85)	68 (41–84)	65 (49–86)	68 (42–84)	68 (41–86)
Gender (male/female)	42/27	21/10	31/21	96/59	190/117
PS (0/1/2/3)	30/31/7/1	15/14/1/1	36/14/1/1	94/52/5/4	175/111/14/7
CA 19-9 before CRT (U/mL)	167 (1–10,093)	286 (1–4687)	139 (1–7054)	187 (0–17,269)	177 (0–17,269)
Tumor size (mm)	27 (12–60)	32 (16–52)	30 (7–76)	37 (13–91)	33 (7–91)
Tumor location (Ph/Pb/Pt)	50/4/15	25/6/0	41/5/6	90/43/22	206/58/43
Chemotherapy (GEM/S-1+GEM)	35/34	14/17	17/35	62/93	128/179

PDAC: pancreatic ductal adenocarcinoma, CRTS: chemoradiotherapy followed by surgery, R: resectable, BR-PV: borderline resectable (superior mesenteric vein/portal vein invasion alone), BR-A: borderline resectable (arterial invasion), LA: locally advanced, PS: Performance status, Ph: pancreatic head, Pb: pancreatic body, Pt: pancreatic tail, GEM: gemcitabine.

**Table 2:** Clinical outcomes of CRTS in the re-evaluated 285 PDAC patients according to resectability groups.

	R n = 62	BR-PV n = 27	BR-A n = 50	LA n = 146	All n = 285
Metastasis within 3 months from initial treatment (among 285 re-evaluated patients)	9.7 (6/62)	11.1 (3/27)	10.0 (5/50)	11.0 (16/146)	10.5% (30/285)
Non-resection at re-evaluation (among 285 re-evaluated patients)	11.3 (7/62)	14.8 (4/27)	22.0 (11/50)	44.5 (65/146)	30.5% (87/285)
Non-resection at laparotomy (among 194 operable patients at re-evaluation)	5.7 (3/53)	0 (0/23)	10.8 (4/37)	16.0 (13/81)	10.3% (20/194)
Resection (among 285 re-evaluated patients)	83.9 (52 */62)	85.2 (23 */27)	70.0 (35 */50)	46.6 (68 */146)	62.5% (178 */285)
R0 resection (among resected patients)	98.0 (48 +/49)	95.5 (21/22)	84.8 (28/33)	62.5 (40 +/64)	81.5% (137 ^+^/168)

* Including four patients with distant metastasis at re-evaluation (two in R and two in BR-A) and six patients with intraabdominal distant metastasis at laparotomy (one in R, one in BR-PV and four in LA); ^+^ Including one patient with metastasis of stomach wall (one in R) and one with paraaortic lymph node metastasis (one in LA); CRTS: chemoradiotherapy followed by surgery, PDAC: pancreatic ductal adenocarcinoma, R: resectable, BR-PV: borderline resectable (superior mesenteric vein/portal vein invasion alone), BR-A: borderline resectable (arterial invasion), LA: locally advanced.
